# Executive Functions May Predict Outcome in Deep Brain Stimulation of Anterior Nucleus of Thalamus for Treatment of Refractory Epilepsy

**DOI:** 10.3389/fneur.2018.00324

**Published:** 2018-05-08

**Authors:** Soila Järvenpää, Eija Rosti-Otajärvi, Sirpa Rainesalo, Linda Laukkanen, Kai Lehtimäki, Jukka Peltola

**Affiliations:** ^1^Department of Neurosciences and Rehabilitation, Tampere University Hospital, Tampere, Finland; ^2^University of Tampere, Tampere, Finland

**Keywords:** deep brain stimulation, refractory epilepsy, anterior nucleus of thalamus, neuropsychological evaluation, patient selection, connectivity, executive functions, seizure

## Abstract

**Background:**

Deep brain stimulation (DBS) of the anterior nucleus of thalamus (ANT) is an emerging treatment option for patients suffering from refractory epilepsy. ANT has extensive connections with hippocampus and retrosplenial cingulum, areas associated mainly with spatial memory and with anterior cingulum which is important in executive functions. As refractory epilepsy is often associated with cognitive decline and neuronal damage, the decreased connectivity between ANT and remote structures might impact on the effects of DBS.

**Objective:**

We hypothesized that the neuropsychological profile could reflect the connectivity of ANT and further predict the efficacy of ANT DBS. We evaluated the cognitive performance of patients with refractory epilepsy with DBS to evaluate whether neuropsychological profiles could reflect the connectivity of ANT and further predict the efficacy of ANT DBS.

**Method:**

Sixteen patients with refractory epilepsy treated with ANT DBS with at least 2 years of follow-up were included in the study. Patients underwent a neuropsychological evaluation as a part of the protocol and their clinical outcome was determined by seizure frequency in the last 6 months compared to baseline. The patients were classified as responders if there was a ≥50% reduction in the frequency of the predominant seizure type, otherwise as nonresponders.

**Results:**

There were 12 responders and 4 nonresponders for ANT DBS treatment in the study population. Nonresponders performed worse than responders in neuropsychological tasks measuring executive functions and attention, such as the Trail-Making Test.

**Conclusion:**

Better executive functions and attention seemed to predict improved clinical outcome after the ANT DBS surgery. Based on our preliminary descriptive findings and the anatomical connectivity hypothesis, we suggest that deficits in executive functions may relate to an inferior outcome. This finding might offer new tools for refining the selection of patients with refractory epilepsy scheduled to undergo ANT DBS surgery. Moreover, it highlights the need for further investigations of neural connectivity in epilepsy.

## Introduction

It has been estimated that there are 50 million people in the world with epilepsy. For more than 30% of these patients, the condition is classified as refractory with continuing uncontrolled seizures despite administration of antiepileptic drugs (AEDs) (1).

Deep brain stimulation (DBS) of the anterior nucleus of thalamus (ANT) has been shown to represent a promising treatment option for patients suffering from refractory epilepsy (2–5). With its numerous connections, ANT can be considered as a crucial node integrating cortical and subcortical information; it lies at a site allowing it to influence the brain’s predisposition to epileptic seizures (4, 6, 7). ANT is linked by white matter tracts to networks regulating amnestic functions and consciousness (circuit of Papez), a hypothetical neuronal circuitry crucial in mediating the formation of memories and emotion (8, 9, 10).

**Figure 1:**
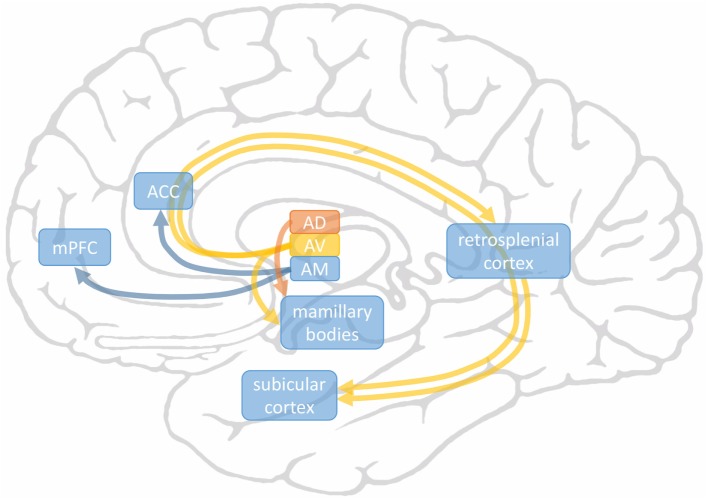
Main connections of anterodorsal (AD), AV, and AM subnuclei of anterior nucleus of thalamus. AM connects to frontal areas medial prefrontal cortex (mPFC) and anterior cingulate cortex (ACC) (in blue). AV is connected to subicular cortex of hippocampus both directly and indirectly *via* the retrosplenial cortex (in yellow). The medial mammillary nucleus receives axons from AV and the lateral mammillary nucleus from AD (in orange).

**Figure 2:**
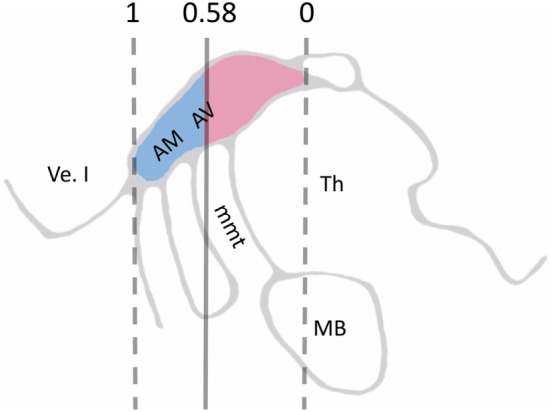
Schematic illustration of visible landmarks in 3T MRI sagittal plane and ANT normalized coordinate system, where the anterior border of ANT is set to 1 and its posterior border to 0 on the *y*-axis. In all, 74% of the deep brain stimulation contacts situated in the anterior side of the cutoff level 0.58 (*blue color*) lead to a favorable outcome to the treatment. By contrast, 83% of the contacts situated posterior to this cutoff level (*red color*) were nonresponders. Abbreviations: AM, anteromedial nucleus of ANT; AV, anteroventral nucleus of ANT; Th, thalamus; Ve. I, lateral ventricle; mmt, mammillothalamic tract; ANT, anterior nucleus of thalamus (11).

Three subnuclei can be functionally distinguished in ANT: anteromedial nucleus of ANT (AM), anterodorsal (AD), and anteroventral nucleus of ANT (AV; also called anterior principal, Apr) nuclei. These are elaborately connected to frontotemporal structures although with different intensities (Figure 1). Anatomically, AM innervates prefrontal areas [medial prefrontal cortex (mPFC); anterior cingulate cortex (ACC)] and is thus considered to play a role in executive functions and attention. AV has connections to hippocampus and is believed to be involved in memory functions. AD is mainly connected to the lateral mammillary nucleus, possibly taking part in mental and spatial navigation. Here, the rostrally situated AM and its frontal connections were a focus of special interest since we recently suggested that a more anterior location of active contact within the ANT complex may be associated with a better outcome after DBS (11) (Figure 2).

As neuropsychological performance has not yet been studied as an outcome predictor in DBS of ANT, the aim of this study was to clarify whether the clinical outcome could be predicted based on the neuropsychological profile prior to or soon after surgery. We have devised an anatomically based hypothesis that performance in executive functions reflects the connectivity remaining between anterior cingulum, prefrontal cortex and ANT and thus has an effect on the outcome of the surgical procedure. Alternatively, severe hippocampal atrophy and memory decline might be associated with decreased connectivity between ANT and hippocampus and potentially a poorer therapeutic effect.

## Materials and Methods

### Patients

We reviewed retrospectively the neuropsychological evaluation reports of patients with refractory epilepsy treated with ANT DBS in Tampere University Hospital between 2011 and 2014. All patients were examined with video-EEG telemetry, 18-F-FDG-PET, and 3T MRI for evaluation if they were suitable for resective surgery. All patients provided written informed consent and the study was approved by the Ethics Committee of Tampere University Hospital.

Out of the 20 patients that underwent ANT DBS surgery and had been evaluated by a neuropsychologist prior to or shortly after the surgery, three of the reports had not been conducted with the latest protocol and were thus unavailable. One patient with a favorable outcome had only been assessed with two tests [Trail-Making Test A (TMT A) and Rey-Osterrieth Complex Figure Test (ROCFT) Copy] that require functional motor abilities; this patient was excluded from the study as being unable to perform the tests reliably because of significant ataxia. Thus, there were 16 patients included in the study.

The 16 patients consisted of 5 women and 11 men. The mean age was 37.9 (SD 10.7) years. The patient population was heterogeneous in terms of etiology, MRI findings, and AED therapy (Table 1). The mean number of AEDs used in combination at the time of the neuropsychological evaluation was 2.8 (SD 0.8). If subdivided by the epilepsy etiology, five patients had encephalitis, six had suffered cortical dysplasia (CD), and five had some unknown etiology. When investigated by scalp EEG, eight had a multifocal epileptic zone, whereas in seven patients, it was localized on one of the hemispheres with one unknown localization. The MRI findings varied from inflammatory lesions, CD, periventricular heterotopia, perisylvian polymicrogyria, hemimegencephalia to normal. Eight of the patients had normal MRI findings.

**Table 1:** Patient demographics as described by age, sex, years of education, etiology of epilepsy, MRI findings, localization of epileptic zone by scalp EEG, antiepileptic drugs (AED), and outcome status defined by at least a 50% reduction in seizure frequency.

Patient	Age	Education (years)	Etiology	MRI	Epileptic zone	AED	Responder
1	31–35	12	Encephalitis	Normal	Multifocal	CBZ, LCM	Yes
2	26–30	9	Encephalitis	Normal	Multifocal	CLB, OXC, ZNS	Yes
3	26–30	12	CD	Bilateral perisylvian polymicrogyria	Multifocal	OXC	Yes
4	51–55	12	Encephalitis	Right parietal and temporal inflammatory lesion	Right temporal	CLB, OXC, ZNS	Yes
5	26–30	12	Encephalitis	Bilateral parietal inflammatory lesion	Multifocal	CLN, PHT	Yes
6	51–55	12	Unknown	Normal	Unknown	LCM, LEV, VPA	Yes
7	36–40	12	CD	Hemimegalencephalia	Right frontal	CLB, OXC, ZNS	Yes
8	31–35	9	CD	Bilateral periventricular heterotopia	Multifocal	CBZ, CLB	Yes
9	36–40	9	CD	Left frontal cortical dysplasia	Left frontal	CLB, LTG, VPA, ZNS	Yes
10	31–35	16	CD	Bilateral periventricular heterotopia	Multifocal	CBZ, LCM, LEV, ZNS	Yes
11	56–60	9	Unknown	Normal	Multifocal	CLB, LCM, LEV, OXC	Yes
12	26–30	12	Unknown	Normal	Right frontal	CLB, LCM, OXC	Yes
13	51–55	12	CD	Bilateral perisylvian polymicrogyria	Right temporal	CBZ, CLB, ZNS	No (dead)
14	21–25	12	Unknown	Normal	Left parietal	CBZ, CLB	No (dead)
15	26–30	9	Encephalitis	Normal	Multifocal	CLB, OXC, TPR	No
16	46–50	12	Unknown	Normal	Right frontal	OXC, VPA, ZNS	No

*CD, cortical dysplasia; CBZ, carbamazepine; CLB, clobazam; CLN, clonazepam; LCM, lacosamide; LEV, levetiracetam; LTG, lamotrigine; OXC, oxcarbazepine; PHT, phenytoin; TPR, topiramate; VPA, valproate; ZNS, zonisamide*.

The follow-up time was at least 2 years. The patients were classified as responders if there had been a ≥50% reduction in frequency of the predominant seizure type in the last 6 months compared to baseline, as suggested previously by Orosz et al. (12). The predominant seizure type was determined by a physician as being the clinically most disabling seizure type, not necessarily the most frequent or the most severe seizure type. According to this classification, there were 12 responders and 4 nonresponders in the study population. The mean age was 37.9 (SD 10.3) years for responders and 37.8 (SD 13.6) years for nonresponders. The mean years of education were the same, i.e., 11.3, in both responders (SD 2.1) and nonresponders (SD 1.5). A subgroup of patients (five responders) was evaluated both prior to and after the surgery.

### Surgery

Surgery was planned using the Surgiplan (Elekta) software. The initial surgical target was defined to be 5–6 mm lateral, 12 mm superior, and 0–2 mm anterior to the midcommissural point. The mammillothalamic tract (mmt) and ANT nucleus were identified individually from the 3T MRI and the surgical target was adjusted accordingly (13). A transventricular trajectory was performed primarily (26/32) with the secondary option being the paraventricular trajectory which was chosen in cases where there were abundant ventricular veins (6/32). Under general anesthesia, the surgery was performed using insertion cannula reaching to 10 mm from the planned target to implant the DBS electrodes (Medtronic 3389). The electrodes were fixed to the skull and connected to extension cables and further to the internal pulse generator (Activa PC, Medtronic) implanted within a subcutaneous pocket in the upper chest area.

The DBS was turned on in the fifth postoperative day. Stimulation was programmed to a cycle of 1 min ON and 5 min OFF with a frequency of 140 Hz and a pulse width of 90 µs. The amplitude was increased to 5 V during the first weeks of stimulation. The initially chosen contacts were changed if necessary due to nonresponsiveness or adverse effects.

### Neuropsychological Evaluation

The neuropsychological evaluation was performed as a part of the DBS protocol in our institution. The median timing for the neuropsychological evaluation was 1 day before the implantation and a mean of 130 days after the implantation (SD 317 days). The following cognitive domains were evaluated: (1) executive functions and attention; (2) memory and learning; (3) language functions; and (4) visual functions. Executive functions and attention were evaluated using the Controlled Oral Word Association Test (COWAT) including semantic (animals) and phonemic (letters P-A-S; a Finnish version of F-A-S) fluency, the Trail-Making Test A and B (TMT A+B), digit span of Wechsler Adult Intelligence Scale III (WAIS-III), and the Stroop Color-Word Interference Test (14). The Rey Auditory Verbal Learning Test (RAVLT) (15) and drawing from memory of ROCFT were used in the evaluation of memory and learning. Total score (total number of words recalled in trials 1 through 5), post-interference recall (number of words recalled after interference), and recognition (number of correctly recognized words in delayed recall) of the RAVLT were used in the analysis. Language functions were evaluated with the WAIS-III’s (16) Similarities. Visual functions were assessed by copying and copying time of the ROCFT (17) and block design of the WAIS-III.

Raw scores of cognitive tests were converted to age-standardized *Z*-scores using published Finnish [WAIS-III; (16)] and international (18) normative data. The scores were first transformed so that a larger value always indicated a better performance. To determine a composite score for each of the four cognitive domains, an average of the *Z*-scores was calculated. In this study, the *Z*-scores were scaled as follows: *Z*-scores equal to or smaller than −3 were considered indicative of a severe deficit; *Z*-scores from −2.99 to −2 were indicative of a moderate deficit; and *Z*-scores from −1.99 to −1 were indicative of a mild deficit. Scores equal to or greater than −0.99 were considered to demonstrate normal performance.

## Results

### Cognitive Performance in Responders and Nonresponders

**Figure 3:**
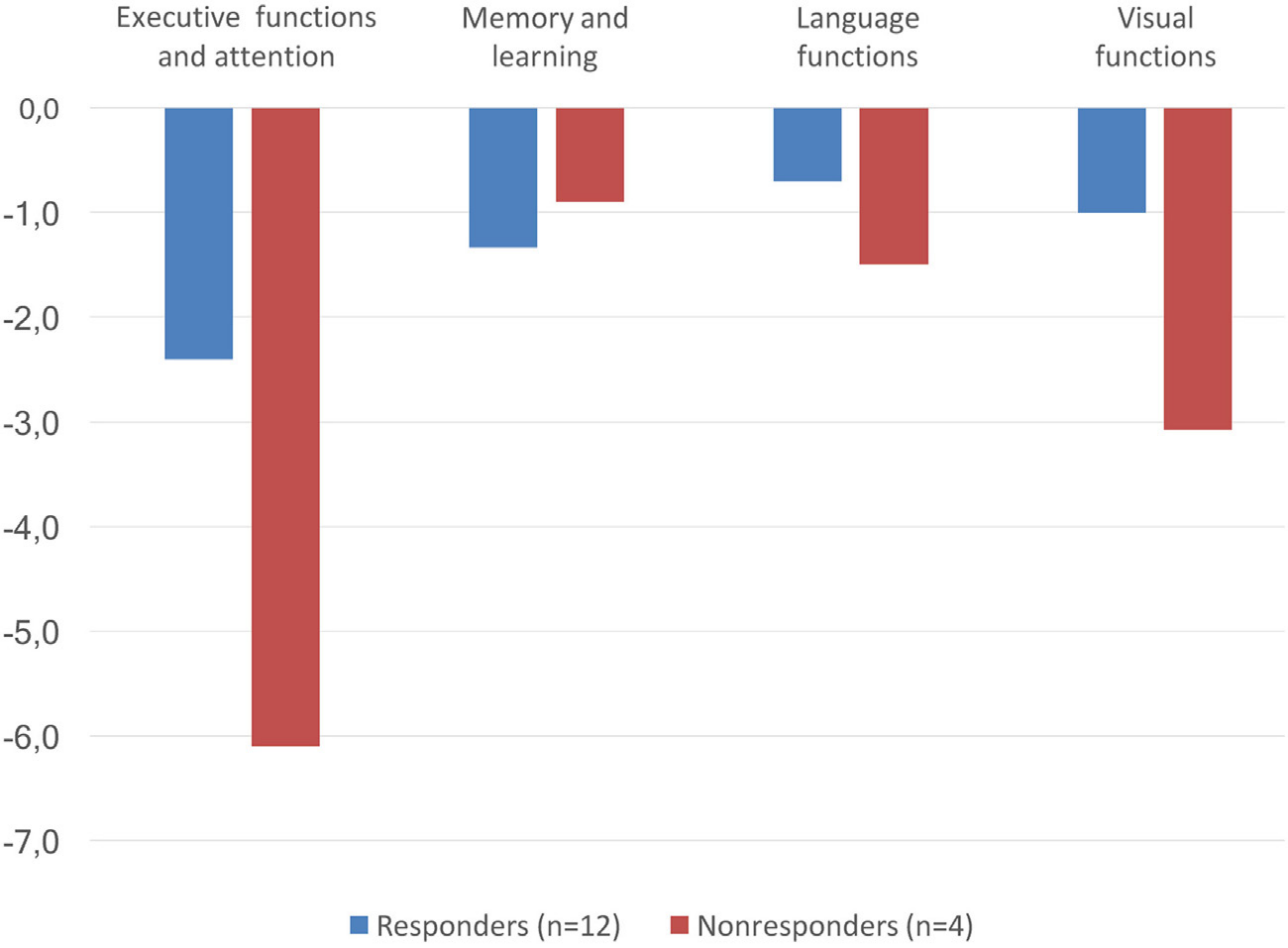
Composite *Z*-scores in different cognitive domains in responders (*n* = 12, in blue) and nonresponders (*n* = 4, in red).

Figure 3 illustrates the performance of responders and nonresponders in different cognitive domains. All tests were not performed by all the patients and some tests were missing one to four values, as seen in Table 3. The cognitive performance remained below normative values in all patients especially in the nonresponders. The performance of the nonresponders was particularly poor in executive functions and attention: the difference in composite *Z*-score was −3.7 (−2.4 for responders and −6.1 for nonresponders). In the responders, composite *Z*-scores were −1.3 for memory and learning, −0.7 for language functions, and −1.0 for visual functions. In the nonresponders, the corresponding composite *Z*-scores were −0.9 for memory and learning, −1.5 for language functions, and −3.1 for visual functions. Four responders and one nonresponder had seizure onset zone in frontal lobe.

The results of cognitive tests for responders and nonresponders are shown in Table 2. With respect to the individual cognitive tests, the difference in performance was most evident in the TMT. In part A, the mean time for responders was 46.0 s (SD 18.9) [−2.3 (2.4) in *Z*-score] whereas it was three times longer in the nonresponders, 138.3 s (113.8) [−12.1 (12.2) in *Z*-score]. Similarly, in part B, the mean time was 153.7 s (60.7) [−4.2 (3.1) in *Z*-score] in the responders and 385.0 s (311.9) [−14.3 (13.9) in *Z*-score] in nonresponders.

**Table 2:** Raw scores (mean, SD) and *Z*-scores (mean, SD) of responders (*n* = 12) and nonresponders (*n* = 4).

	Raw score for responders (SD)	Raw score for nonresponders (SD)	*Z*-score for responders (SD)	*Z*-score for nonresponders (SD)
**Executive functions and attention**				
Wechsler Adult Intelligence Scale III (WAIS-III)				
Digit span	9.9 (3.3)	6.3 (2.1)	−1.3 (0.9)	−2.1 (0.5)
Controlled Oral Word Association Test				
Animal fluency	12.4 (5.3)	7.5 (2.1)	−2.3 (1.1)	−3.0 (0.6)
Word fluency	23.7 (12.2)	14.0 (15.6)	−1.8 (1.1)	−2.7 (1.5)
Trail-Making Test				
Part A (s)	46.0 (18.9)	138.3 (113.8)	−2.3 (2.4)	−12.1 (12.2)
Part B (s)	153.7 (60.7)	385.0 (311.9)	−4.2 (3.1)	−14.3 (13.9)
Stroop				
Color-word	27.3 (8.9)	21.5 (12.0)	−2.4 (1.1)	−2.4 (1.7)

**Memory and learning**				
Rey-Osterrieth Complex Figure Test (ROCFT)				
Immediate recall	13.1 (6.1)	13.0 (5.7)	−1.8 (1.1)	−1.8 (0.2)
Rey Auditory Verbal Learning Test				
Total recall	40.5 (9.2)	44.3 (0.6)	−1.6 (1.0)	−1.1 (0.1)
Post-interference recall	6.4 (3.4)	9.0 (1.4)	−1.6 (1.2)	−0.8 (0.6)
Recognition	13.3 (2.0)	14.0 (1.4)	−0.3 (1.1)	0.1 (1.2)

**Language functions**				
WAIS-III				
Similarities	18.6 (5.1)	10.8 (4.9)	−0.7 (0.9)	−1.5 (0.7)

**Visual functions**				
ROCFT				
Copying time (s)	325.1 (140.4)	540.3 (106.0)		
Copy	32.8 (2.0)	24.7 (15.3)	−1.2 (1.3)	−4.6 (6.3)
WAIS-III				
Block design	32.9 (10.4)	19.5 (9.3)	−0.8 (0.7)	−1.6 (0.6)

The severity of cognitive dysfunction in different cognitive tests is presented in Table 3. As measured by the *Z*-score, normal performance was not achieved in the nonresponders in any of the tests evaluating executive functions and attention. The deteriorated performance was especially evident in all of the nonresponders in the TMT. Some responders were also severely impaired in executive functions and attention, but their performance was distributed more evenly into different degrees of severity. In addition, in tests of visual functions, the performance of nonresponders was more often severely impaired in comparison with the responders. In memory, learning, and language functions, the performance of the patients was closer to normal and differences between the groups were not as evident as in the other domains.

**Table 3:** Severity of cognitive dysfunction based on *Z* values in responders and nonresponders.

Responders (***n*** = 12)	Normal (*Z* > −1)	Mild (−2 < *Z* ≤ −1)	Moderate (−3 < *Z* ≤ −2)	Severe (*Z* ≤ −3)
**Executive functions and attention**				
WAIS-III				
Digit span	3 (25.0%)	5 (41.7%)	4 (33.3%)	0 (0%)
COWAT				
Animal fluency	1 (8.3%)	5 (41.7%)	2 (16.7%)	4 (33.3%)
Word fluency	3 (25.0%)	4 (33.3%)	2 (16.7%)	3 (25.0%)
Trail-Making Test (TMT)				
Part A (s)	5 (41.7%)	1 (8.3%)	1 (8.3%)	5 (41.7%)
Part B (s)	1 (8.3%)	2 (16.7%)	1 (8.3%)	8 (66.7%)
Stroop				
Color-word^c^	0 (0%)	4 (44.4%)	3 (33.3%)	2 (22.2%)
**Memory and learning**				
ROCFT				
Immediate recall	2 (16.7%)	4 (33.3%)	4 (33.3%)	2 (16.7%)
RAVLT				
Total recall^a^	3 (27.3%)	5 (45.5%)	2 (18.2%)	1 (9.1%)
Post-interference recall^a^	5 (45.5%)	0 (0%)	5 (45.5%)	1 (9.1%)
Recognition^d^	6 (75.0%)	1 (12.5%)	1 (12.5%)	0 (0%)
**Language functions**				
WAIS-III				
Similarities^a^	6 (54.5%)	4 (36.4%)	1 (9.1%)	0 (0%)
**Visual functions**				
ROCFT				
Copy	6 (50.0%)	2 (16.7%)	4 (33.3%)	0 (0%)
WAIS-III				
Block design^a^	6 (54.5%)	3 (27.3%)	2 (18.2%)	0 (0%)
**Nonresponders (***n*** = 4)**				
**Executive functions and attention**				
WAIS-III				
Digit span	0 (0%)	2 (50.0%)	2 (50.0%)	0 (0%)
COWAT				
Animal fluency^b^	0 (0%)	0 (0%)	1 (50.0%)	1 (50.0%)
Word fluency^b^	0 (0%)	1 (50.0%)	0 (0%)	1 (50.0%)
TMT				
Part A (s)^a^	0 (0%)	0 (0%)	0 (0%)	3 (100%)
Part B (s)^a^	0 (0%)	0 (0%)	0 (0%)	3 (100%)
Stroop				
Color-word^b^	0 (0%)	1 (50.0%)	0 (0%)	1 (50.0%)
**Memory and learning**				
ROCFT				
Immediate recall^b^	0 (0%)	2 (100%)	0 (0%)	0 (0%)
RAVLT				
Total recall^a^	1 (33.3%)	2 (66.7%)	0 (0%)	0 (0%)
Post-interference recall^b^	1 (50.0%)	1 (50.0%)	0 (0%)	0 (0%)
Recognition^b^	2 (100%)	0 (0%)	0 (0%)	0 (0%)
**Language functions**				
WAIS-III				
Similarities	1 (25.0%)	2 (50.0%)	1 (25.0%)	0 (0%)
**Visual functions**				
ROCFT				
Copy^a^	1 (33.3%)	1 (33.3%)	0 (0%)	1 (33.3%)
WAIS-III				
Block design^d^	1 (25.0%)	1 (25.0%)	2 (50.0%)	0 (0%)

*Values shown are number of patients (percentages in parentheses)*.

*WAIS-III, Wechsler Adult Intelligence Scale III; COWAT, Controlled Oral Word Association Test; RAVLT, Rey Auditory Verbal Learning Test; ROCFT, Rey-Osterrieth Complex Figure Test*.

*^a^One missing value*.

*^b^Two missing values*.

*^c^Three missing values*.

*^d^Four missing values*.

### Preoperative and Postoperative Cognitive Performance in a Subgroup of Patients

**Figure 4:**
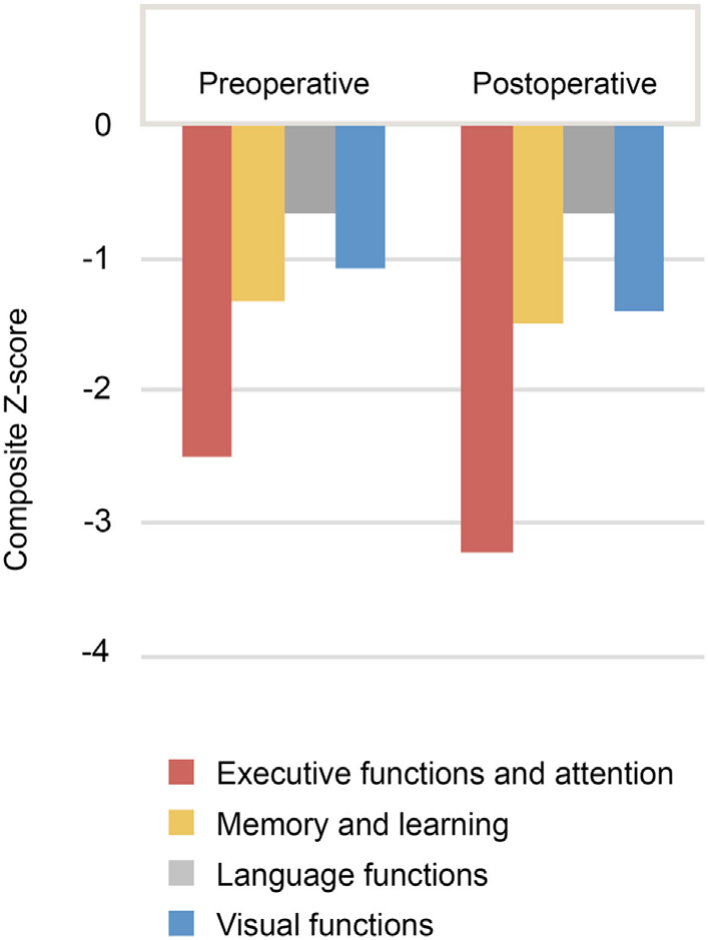
Composite *Z*-scores in different cognitive domains for patients in whom both preoperative and postoperative neuropsychological evaluations were available (*n* = 5).

A subgroup of patients (*n* = 5) underwent both prior and post-surgical evaluation. As can be seen in Figure 4, there was no significant difference in the patients’ cognitive performance between the preoperative and postoperative evaluations.

## Discussion

The concept of ANT DBS as treatment of refractory epilepsy is relatively new. Previous trials examining its potential for reducing seizure frequency have been promising. However, the reduction in the seizure rate between patients varies extensively but the reasons for this variation remain a mystery (2–5). Only a few studies have been published about factors predicting outcome; these have considered the influence of surgical and imaging techniques. It has found that DBS contacts located in the anterior aspect of ANT and the use of a transventricular approach during surgery have led to more a favorable outcome (11, 19). As the anterior aspect of ANT seems to be the most appropriate location for DBS electrodes, we hypothesized that correct functioning of the anatomical connections associated with anterior parts of ANT would associate with a favorable outcome. Here, we have evaluated the functioning of these neuronal connections by examining neuropsychological performance in different cognitive domains and compared this to treatment outcome.

In our study, the patients’ performance in the neuropsychological tests was mainly below average in all cognitive functions. This finding is in line with one known feature of epilepsy as a disease, i.e., it causes a cognitive decline (20). Better executive functions and attention seemed to associate with better clinical outcome, possibly reflecting the level of connectivity of the ANT to the frontal cortex. In the individual cognitive tests, the age-standardized *Z*-score was notably better in the TMT in responders than in nonresponders. Less evident differences were detected in other cognitive domains. The performance of responders was better than that of nonresponders also in the visual functions domain. It could be argued that this finding also emphasizes the effect of the executive functions on the outcome, because the tests included in the visual functions domain (ROCFT and block design) also require executive functions. There were severe deficits in executive functions and attention as well as in visual functions in the nonresponders. Instead among responders, the deficits were moderate with respect to executive functions and attention and even only mild for visual functions. Memory and learning—functions localized in the limbic circuit—seemed to display no association with the outcome.

Several of the cognitive domains evaluated in our study, i.e., executive functions, attention, memory, and learning, have been associated with connections to ANT. The AM subnucleus is connected to frontal areas, i.e., mPFC and ACC, which are known to participate in executive functions and attention (21, 22). Memory and learning have been postulated to form the so-called Papez circuit of which ANT is a part (8–10).

In more detail, ANT is located in the anterior aspect of the dorsal thalamus, bordered from both sides by branches of internal medullary lamina (23). Significant projections to ANT originate from mmt, entorhinal cortex, and adjoining hippocampal structures. ANT lies within the Papez circuit that consists of thalamus, sensory cortex, hypothalamus, cingulate cortex, and hippocampus. In theory, ANT will be receiving information from hypothalamus *via* mmt, directing it further through the internal capsule to cingulate cortex, a structure that integrates these emotional stimuli with the information arriving from the sensory cortex. Cingulate cortex further directs information back to the hypothalamus *via* the cingulum. The information is passed onward along the fornix to the entorhinal cortex of the hippocampus, which links the circuit to the limbic system. This circuit connects ANT frontally to structures mediating executive functions and temporally to functions including learning and memory (8, 9, 10). ANT DBS has been observed to activate several structures in the prefrontal area in a study conducted with anesthetized swine (24).

The existing data suggest that some alterations in neuropsychological domains might occur later after the initiation of ANT DBS treatment, in fact several studies detected no consistent changes in cognitive functions immediately after the surgery. Five of our patients were examined prior to and after surgery with no significant change in patients’ cognitive performance being detected between the two time points. In recent studies, a cognitive improvement has been observed in patients with ANT DBS in verbal fluency and delayed verbal memory, whereas a deterioration of response inhibition and increased emotional reactivity to threat has been reported (25, 26). No significant change in executive functions, information processing, and general abilities, and furthermore, no correlation has been found between the reduction of seizure frequency and neuropsychological composites (25). A frequency of 13% has been reported for the subjective worsening of memory at 3 months after ANT DBS surgery (2). This effect was, however, transitory and had disappeared at the 5 years’ follow-up after surgery. In a further study, subjectively reported memory problems were not observed in the neuropsychological evaluation, in fact, the tested memory functions seemed to show a slight objective improvement over time (27). A gradual improvement has been noted in cognition and mood as well as a deterioration in expressive language in the 5-year follow-up, however, there was no difference between control and stimulated groups at the end of the 3-month blinded phase. Furthermore, no differences between responders and nonresponders have been reported. Based on these previous findings and our own data, it seems that the neuropsychological profile remains relatively stable soon after the surgery.

Our study was carried out retrospectively and has some limitations. First, the patient population is heterogenic in terms of etiology and MRI findings. Second, the sample size was relatively small, consisting of only 16 patients, which did not permit extensive statistical comparisons between the groups. Many of the calculations have been estimated as mean values and this might have a distorting impact on some results. Third, we included neuropsychological evaluations made before and after the surgery. Based on previous studies, we assumed that the neuropsychological performance would not be significantly affected by the ANT DBS treatment. Fourth, our study findings are based on indirect evidence measuring only the contribution of individual variables and do not segregate whether executive function performance is independent predictor of worse outcome or just reflects the role of other latent variables not included in the statistical analysis. Etiopathogenesis of cognitive decline in epilepsy is multifactorial and worse performance in executive functions might result from diffused dysfunction of prefrontal lobe. Hence, the relationship between neuropsychological performance and the connectivity of ANT to the frontal cortex is not unambiguous. Frontal lesions and epileptic discharges on frontal cortex might indicate the seizure onset zone to be on prefrontal cortex and patients like this could be modulated more by ANT DBS due to direct connectivity between ANT and prefrontal cortex. On the other hand, frontal epilepsy might decrease prefrontal connectivity of ANT and therefore lead to worse outcome in ANT DBS. In our study, there were four responders and one nonresponder with frontal epilepsy. This suggests that the correlation between executive function performance and seizure reduction outcome requires further investigation.

In conclusion, better executive functions and attention seemed to predict a better clinical outcome of the ANT DBS treatment. Based on our preliminary findings and the anatomical connectivity hypothesis, we suggest that deficits in executive functions may relate to poorer outcome, possibly due to decreased prefrontal connectivity in the ANT. This finding might offer a potential foundation for further research to help refine the selection of patients with refractory epilepsy who are candidates for ANT DBS surgery, a subject that has not been extensively studied. The implantation of DBS electrodes is a minimally invasive neurosurgical procedure and reversible in nature, but like all surgical procedures, it entails some risks and should only be performed after thorough consideration. Our hypothesis forming study aims to introduce the possible significance of functional anatomical connections in the ANT to help achieve favorable treatment results. Moreover, it encourages future research on neuropsychological evaluation as a tool for patient selection, highlighting the need for further investigation of possible confounding factors, such as the effect of seizure load and seizure type, presence or absence of episodes of convulsive status and other variables.

## Ethics Statement

The study was approved by the Ethics Committee of Pirkanmaa Hospital District. Written informed consent was obtained from each of the patients.

## Author Contributions

SJ and ER-O contributed to study conception, neuropsychological outcome evaluations, and drafted the manuscript. SR contributed to patient follow-up and analysis and drafted the manuscript. LL contributed to the neuropsychological outcome evaluations. KL contributed to study conception and drafted the manuscript. JP contributed to study conception, neurological follow-up, and drafted the manuscript.

## Conflict of Interest Statement

KL and JP have received speaker and consultation fees from Medtronic. The other authors declare that the research was conducted in the absence of any commercial or financial relationships that could be construed as a potential conflict of interest. The reviewer LR and handling Editor declared their shared affiliation.
